# Glycoprotein G (gG) production profile during infectious laryngotracheitis virus (ILTV) infection

**DOI:** 10.1371/journal.pone.0219475

**Published:** 2019-08-21

**Authors:** Jorge Bendezu, Sandra Morales Ruiz, Ricardo Montesinos, Ricardo Choque Guevara, Aldo Rojas-Neyra, Katherine Pauyac-Antezana, Manolo Fernández-Díaz

**Affiliations:** Laboratorios de Investigación y Desarrollo—FARVET, Chincha Alta, Ica, Peru; Institute of Molecular Biology, Academia Sinica, TAIWAN

## Abstract

Glycoprotein G (gG) is a conserved protein, and it has been described as a chemokine-binding protein in most members of the alphaherpesviruses. In case of the infectious laryngotracheitis virus (ILTV), an alphaherpesvirus that infects chickens, this protein is a virulence factor that plays an immunomodulatory role in the chicken immune response. Nevertheless, the gG production profile during ILTV infection has not yet been studied. In this study, we developed monoclonal antibodies in order to determine the gG production profile during ILTV infection in chicken hepatocellular carcinoma (LMH) cell cultures as well as embryonated specific-pathogen-free (SPF) chicken eggs and SPF chickens using a sandwich enzyme-linked immunosorbent assay (ELISA). Despite the fact that inoculated LMH cell cultures showed an increase in both gG production and viral genome copy number up to 96 h after inoculation, we observed that gG production started earlier than the increase in viral genome copy number in ILTV infected embryonated SPF chicken eggs. Likewise, a gG production peak and an increase of viral genome copy number was observed prior to the appearance of clinical signs in infected SPF chickens. According to the production profiles, gG was also produced quite early in eggs and chickens inoculated with ILTV. These findings contribute to the knowledge of the gG role during the ILTV infection as a virulence factor.

## Introduction

Infectious laryngotracheitis (ILT) is an upper respiratory avian disease that causes important economic losses in the poultry industry [1]. This disease is caused by the *Gallid alphaherpesvirus type 1*, also known as the infectious laryngotracheitis virus (ILTV) [2].

The ILTV genome, a linear double-stranded DNA, is approximately 150 kbp long and encodes 80 predicted ORFs [3]. Many ILTV proteins have been described in detail [4] and only one of them known as glycoprotein G (gG), encoded by the US4 gene [5], has been reported to be secreted [6, 7] Two properties have been attributed to gG, including chemokine binding [6] and modulation of chicken cytokine transcription [8].

As a viral chemokine-binding protein (vCKBP), gG is a virulence factor able to bind host chemokines in order to prevent migration of specialized immune cells, facilitating viral evasion of the host immune response [6, 9]. As a chicken cytokine transcription modulator, gG can regulate RNA transcript levels of cytokines such as CXCLi1, CXCLi2, CXCL12, and CXCL14 in tracheal organ cultures as well as CXCLi1 and CXCLi2 in blood-derived monocytes [8].

Similar to ILTV gG, gGs of mammal-infecting alphaherpesviruses, such as equine herpesvirus 1 (EHV-1), bovine herpesvirus 1 and 5 (BHVs 1 and 5), felid herpesvirus 1 (FeHV-1), and pseudorabies virus (PRV), have been associated with chemokine activity inhibition [10]. Despite all available knowledge, no study has been carried out to determine the production profile of this important protein during infection as a virulence factor.

Particularly, in the case of ILTV, the identification of ILTV gG from cell cultures was based on either gene expression using RT qPCR [11], northern blot [5], or protein detection using western blot [5, 7, 12, 13]. The aim of this study was to determine ILTV gG production profiles during infection in cell cultures as well as in embryonated specific-pathogen-free (SPF) chicken eggs and SPF chickens using monoclonal antibodies. The profiles were used to evaluate the relationship between this virulence factor and indicators of infection such as the number of viral genome copies and clinical signs.

## Materials and methods

### Cell lines and viruses

The *Spodoptera frugiperda* (Sf9) cell line (Cat. No. B82501; Thermo Fisher Scientific, Carlsbad, CA, USA) was cultivated as a suspension culture at 27°C in Ex-cell 420 serum-free medium for insect cells (Sigma Aldrich Co., St Louis, Missouri, USA). The chicken hepatocellular carcinoma (LMH) cell line (Cat. No. 601411; CLS cell lines service GmbH, Eppelheim, Germany) was cultivated as an adherent culture at 37°C with 5% CO_2_ in Dulbecco’s modified Eagle medium (DMEM) F12 (HyClone, Logan, UT, USA) supplemented with 5% heat-inactivated fetal bovine serum (FBS).

The Peruvian ILTV strain VFAR-043 was isolated from an outbreak occurred on a farm in Chincha Alta, Peru [14] and cultivated in LMH cells following a previously reported method [13]. The Newcastle disease virus (LaSota strain) and the avian metapneumovirus (SHS-FAR strain) were cultivated following published procedures [15], and the infectious bronchitis virus (Q1-type strain) was propagated onto embryonated SPF chicken eggs according to a previous study [16].

### Generation of ILTV-gG expressing recombinant baculovirus

**Fig 1:**
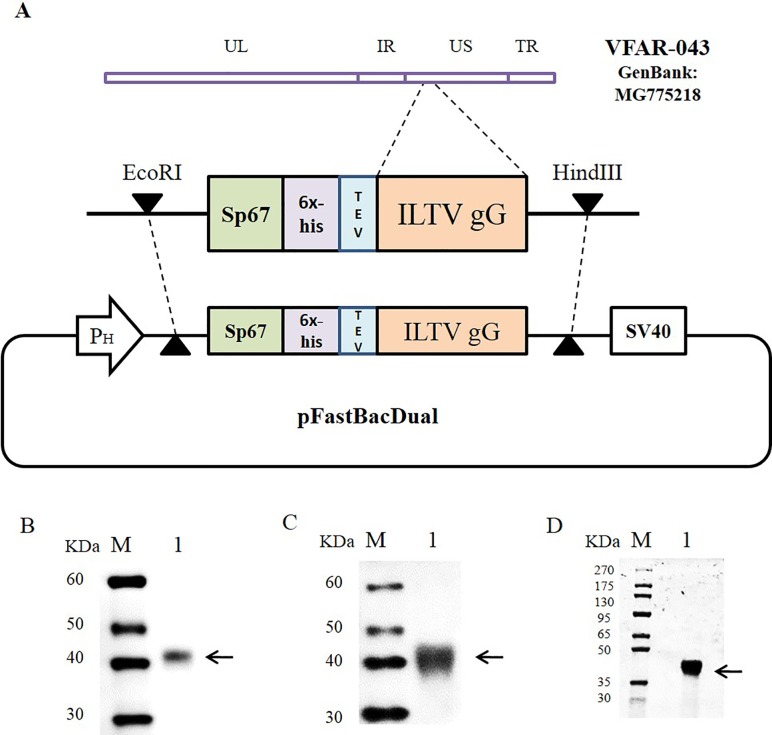
Expression of the ILTV gG recombinant protein using baculovirus. (A) Strategy for cloning ILTV gG from the VFAR-043 strain into pFastBacDual. (B) Western blot detection of secreted ILTV gG from infected Sf9 insect cell supernatants using anti-6x-His tag antibodies. (C) Western blot detection of secreted ILTV gG from infected Sf9 insect cell supernatants using serum from ILTV-infected chicken (Charles Rivers Laboratories). (D) SDS-PAGE detection of purified ILTV gG by Coomassie blue staining. Lane M: Molecular weight marker; Lane 1: ILTV gG recombinant protein. Black arrows indicate the ILTV gG recombinant protein.

The full-length sequence of the gG gene was extracted from a published ILTV genome sequence of VFAR-043 (GenBank accession number: MG775218) and optimized for extracellular insect cell expression. Briefly, the first eighty-seven nucleotides (from 5’ to 3’ direction) were replaced by the following sequences in the order they appear: (i) one-hundred-fourteen nucleotides corresponding to the insect cell signal peptide 67 (Sp67) [17], (ii) eighteen nucleotides corresponding to the 6x-His tag sequence and (iii) twenty-one nucleotides corresponding to the Tobacco Etch Virus (TEV) cleavage site sequence. The resulted sequence flanked by EcoRI and HindIII restriction sites was chemically synthesized (GenScript Laboratories, Piscataway, NJ, USA) and subcloned into pFastBacDual (Fig 1A). Competent *E*. *coli* DH10Bac transformation, transfection, quantification and amplification of the recombinant baculovirus stock were carried out according to the manufacturer's instructions [18].

### Insect cell culture for recombinant ILTV gG production

Fifty milliliters of a Sf9 insect cell culture, seeded at 2 × 10^6^ cells/mL, was infected with the recombinant baculovirus at a multiplicity of infection (MOI) of 2. One-milliliter samples were collected every day after infection for 5 days and centrifuged at 1,000 x g for 10 min at room temperature (RT). The culture supernatant was filtered using a 0.2 μm filter and stored at 4°C for further evaluation.

The ILTV gG recombinant protein production was scaled-up to a three-liter culture using a Biostat B plus bioreactor (Sartorius, Göttingen, Germany) following previously reported parameters and conditions [19]. The cell culture was clarified and concentrated using tangential filtration Sartoflow Advanced equipment (Sartorius, Göttingen, Germany). The obtained retentate was used for protein purification.

### ILTV gG recombinant protein purification

ILTV gG recombinant protein purification was performed in two steps: (i) immobilized-metal affinity chromatography (IMAC) using a HisTrap excel affinity column (1.6 × 2.5 cm) (GE Healthcare, Uppsala, Sweden) and (ii) anion-exchange chromatography (AEX) using a Foresight Nuvia Q column (0.8 × 10 cm) (Bio-Rad Laboratories, Hercules, CA, USA). Both purification processes were performed at RT using the ÄKTA Pure system (GE Healthcare, Uppsala, Sweden). For IMAC, the retentate was incubated with a non-ionic surfactant, nonidet P-40 at 2% (v/v) (Sigma Aldrich Co., St Louis, Missouri, USA), for three h. Briefly, 8 column volumes (CV) of equilibrium buffer (20mM phosphate buffer, 0.5M NaCl [pH 7.5]) were applied to the column before the sample, and then 12 CV of washing buffer (20mM phosphate buffer, 0.5M NaCl, 10mM imidazole, 2% nonidet P-40 [pH 7.5]) were used to remove unbound proteins. Protein elution was performed in a linear gradient (0–55%) using 8 CV of elution buffer (20mM phosphate buffer, 0.5M NaCl, 0.5M imidazole [pH 7.5]). Protein fractions were collected and analyzed by SDS-PAGE under reducing conditions. Then, desalting was performed using a Spin-X UF-20 5K WMCO concentrator (Corning, Nueva York, NY, USA) and phosphate-buffered saline (PBS) at pH 7.4.

For AEX, the desalted protein was diluted three times using equilibrium buffer (20mM Phosphate Buffer [pH 5.5]). Briefly, 5 and 10 CV of equilibrium buffer were used for column equilibration and washing, respectively. Protein elution was performed with a linear gradient (0–100%) using 12 CV of elution buffer (20mM Phosphate Buffer, 1M NaCl [pH 5.5]). Protein fractions were collected and analyzed by SDS-PAGE in reducing conditions. The desalting process was performed as described above. Finally, the purified recombinant protein was quantified using the Bradford assay (Merck KGaA, Darmstadt, Germany).

### Generation of hybridomas and monoclonal antibody production

The purified ILTV gG recombinant protein was subjected to TEV digestion. As a result, we obtained a tag-free ILTV gG recombinant protein, which was used for BALB/c mice immunization in order to obtain hybridomas. The protein digestion, as well as the generation of hybridomas (clones) including BALB/c mice supply, was performed by GenScript Laboratories (Piscataway, NJ, USA). Tag-free ILTV-gG recombinant protein-reactive clones were selected by ELISA assay and isotyped. All positive clones were sent to FARVET Laboratories for monoclonal antibody production according to a previously published methodology (15). Hybridoma culture supernatants were used for antibody purification.

### Monoclonal antibody purification

The culture supernatant from each hybridoma clone was centrifuged for 10 min at 4°C at 13,000 × *g* and filtered using a 0.22 μm filter. The chromatographic runs were performed at RT using an affinity HiTrap rProtein A FF column (0.7 cm × 2.5 cm) (GE Healthcare, Uppsala, Sweden) and an ÄKTA Pure system (GE Healthcare, Uppsala, Sweden). The column was equilibrated using 8 CV of equilibrium buffer (20mM sodium phosphate buffer, 3M NaCl, [pH 7.0]), then the filtered culture supernatant was loaded into the column. The column was washed with 12 CV equilibrium buffer to remove unbound compounds. Finally, monoclonal antibodies were eluted using 10 CV elution buffer (0.1M sodium citrate, [pH 3.5]) in a step gradient. Antibody fractions were collected and analyzed by SDS-PAGE in reducing and non-reducing conditions. Then, desalting was performed using a Spin-X UF-20 50K WMCO concentrator (Corning, Nueva York, NY, USA) and PBS (pH 7.4). Finally, the purified monoclonal antibodies were quantified using a Bradford assay (Merck KGaA, Darmstadt, Germany).

### Sodium dodecyl sulfate polyacrylamide gel electrophoresis (SDS-PAGE)

Protein eluates were diluted in either reducing or non-reducing 5X Laemmli sample buffer, heated at 95ºC for 4 min and separated using a Mini-Protean vertical electrophoresis system (Bio-Rad Laboratories, Hercules, CA, USA) in 4–20% SDS PAGE gels. Gels were stained with Coomassie blue overnight at RT and unstained with acetic acid: methanol: water solution (1:3:6) (v/v).

### Western blot assay

The purified ILTV gG recombinant protein, which was two-fold serially diluted from 1000 ng to 15.6 ng, and the infected Sf9 insect cell supernatants were electrophoretically separated by SDS-PAGE under either reducing or non-reducing conditions and transferred to nitrocellulose membranes using an e-blot device (GenScript Laboratories, Piscataway, NJ, USA). The membranes were blocked with 5% (w/v) BSA in PBS at pH 7.4 and incubated for 1 h at RT. Membranes were washed three times over 30 min with Tris-buffered saline (TBS) containing 0.1% (v/v) Tween 20 (TBS-0.1%) and incubated for 1 h at RT with either each purified monoclonal antibody (1μg/mL), or serum from ILTV-infected chickens (Cat. No. 10100470; Charles Rivers, Wilmington, MA, USA) (1:1000 dilution), or an anti-6x-His tag antibody conjugated to horseradish peroxidase (HRP) (0.5 μg/mL) (Cat. No. A00612; GenScript Laboratories, Piscataway, NJ, USA). Membranes were washed three times with TBST-0.1% and incubated in 1% (w/v) BSA with either goat anti-mouse IgG antibody conjugated to HRP (Cat. No. A00160; GenScript Laboratories, Piscataway, NJ, USA) or goat anti-chicken IgY antibody conjugated to HRP (Cat. No. A00165; GenScript Laboratories, Piscataway, NJ, USA) at 1:5000 and 1:2000 dilutions, respectively. Finally, the membranes were washed three times with TBST-0.1%, incubated with radiance (Azure Biosystems, Dublin, CA, USA) as a substrate and revealed with a CCD camera (Azure Biosystems, Dublin, CA, USA).

### Immunofluorescence of Sf9 cells

Sf9 cells were seeded in a 24-well plate at a density of 2.5×10^5^ cells per well and infected with recombinant baculovirus at an MOI of 4. After 72 h of infection, the cells were fixed with methanol: acetone solution (1:1) at -20°C for 5 min. Fixed cells were washed three times with Dulbecco’s phosphate buffered saline (DPBS) and blocked with 5% (w/v) BSA (Sigma Aldrich Co., St Louis, MO, USA) in DPBS for 60 min at RT. Then, each purified monoclonal antibody diluted in DPBS (2 μg/mL) was added to each well and incubated for 60 min at RT. Each well was washed three times with DPBS and incubated with goat anti-mouse IgG antibody conjugated to Alexa Fluor 594 (Cat. No. ab150120; Abcam, Cambridge, MA, USA) at a concentration of 2 μg/mL for 60 min at RT. After three washing steps, fluorescence microscopy was performed using a fluorescence inverted microscope AXIO Observer A1 (Zeiss, Oberkochen, Germany). Uninfected Sf9 cells were used as negative controls.

### Inmmunofluorescence of LMH cells

LMH cells were seeded in a 24-well plate at a density of 4 × 10^5^ cells per well and infected with the VFAR-043 strain at an MOI of 0.01 for 1 h. Culture medium was removed from each well, and 1% low melting point agarose (Thermo Fisher Scientific, Waltham, MA, USA) in DMEM F12 supplemented with 5% FBS and 50 mg/mL gentamicin was added. After 48 h of infection, the cells were fixed with 4% (v/v) paraformaldehyde solution in DPBS for 4 h at RT and the agarose was removed. Fixed cells were washed three times with DPBS and permeabilized with 0.05% (v/v) Triton X-100 in DPBS for 10 min at RT. Wells were washed three times with DPBS and blocked with 5% (w/v) BSA (Sigma Aldrich Co., St Louis, Missouri, USA) in DPBS for 60 min at RT. Next, dual staining was carried out adding a monoclonal antibody against ILTV gG and a rabbit anti-β-tubulin III antibody (Cat. No. A01203; GenScript laboratory, Piscataway, NJ, USA) at 2 μg/mL. Both antibodies were incubated for 60 min at RT. After washing three times with DPBS, the cells were incubated with goat anti-mouse IgG conjugated to Alexa Fluor 594 (Cat. No. ab150120; Abcam, Cambridge, MA, USA) and donkey anti-rabbit IgG conjugated to Alexa Fluor 488 (Cat. No. ab181346; Abcam, Cambridge, MA, USA) at 2 μg/mL. After three washing steps, nuclear staining was performed with DAPI mounting solution (Abcam, Cambridge, MA, USA). Fluorescence microscopy was carried out in a fluorescence inverted microscope AXIO Observer A1 (Zeiss, Oberkochen, Germany). Uninfected LMH cells were used as negative controls.

### Polyclonal antibody production

Tag-free ILTV gG recombinant protein was used for immunization of two hens. Eggs were collected daily for further immunoglobulin Y (IgY) purification. The immunization and the IgY purification were carried out at Gallus Immunotech Inc. (Fergus, Ontario, Canada). The chicken IgY polyclonal antibody was only used for the sandwich ELISA assay development.

### Indirect ELISA assay

For estimation of the optimal antibody reactivity concentration, MaxiSorp 96-well microplates (Sigma Aldrich Co., St Louis, Missouri, USA) were coated with 100 μL of ILTV gG recombinant protein (1μg/mL) in carbonate/bicarbonate buffer (pH 9.5) and incubated at 4°C overnight. On the following day, wells were washed three times with PBS containing 0.05% (v/v) Tween 20 (PBS-T 0.05%) and blocked with 5% (w/v) BSA (Sigma Aldrich Co., St Louis, Missouri, USA) in PBS-T 0.05% for 1 h at RT. After three washing steps, monoclonal antibodies were two-fold serially diluted (from 10 μg/mL to 0.0195 μg/mL) in 1% BSA (w/v) and added to each well. The microplates were incubated for 1 h at 37°C and washed three times with PBS-T 0.05%. One-hundred microliters of diluted goat anti-mouse IgG antibody conjugated to HRP (1:30 000) (Cat. No. A00160; GenScript laboratory, Piscataway, NJ, USA) was added into the wells and incubated at 37°C for 1 h. The microplates were washed three times and 100 μL of 3,3’,5,5’-tetramethylbenzidine (TMB) substrate (Sigma Aldrich Co., St Louis, Missouri, USA) was added. Then, the microplates were incubated at RT for 15 min. The reaction was stopped by adding 50 μL of 2N H_2_SO_4_ and the microplates were read at 450 nm using an EON microplate reader (Biotek, Winooski, VT, USA).

For the limit of detection of monoclonal antibodies, we used the same procedure mentioned above, but we employed a two-fold serial dilution (from 0.625 μg/mL to 0.005 μg/mL) of the ILTV-gG recombinant protein for microplate coating and the monoclonal antibody concentrations selected from the previous evaluation. A similar procedure was performed to evaluate the specificity of monoclonal antibodies, but we also employed the Newcastle disease virus, the avian metapneumovirus, and the infectious bronchitis virus, in addition to the ILTV gG recombinant protein, as positive controls.

### Determination of gG production in three biological systems inoculated with VFAR-043 strain

#### LMH cell cultures (*in vitro*)

Ten 75 cm^2^-cell culture flasks containing 8 x 10^6^ LMH cells at confluency of 70% were infected with the VFAR-043 strain at a MOI of 0.02 according to a previous study (15). Two flasks were harvested daily from 24 to 120 h after inoculation. The harvested cell cultures were centrifuged at 10,000 × *g* for 30 min at 4°C. The culture supernatants were filtered using a 0.22μm filter and diluted 1:2 in 1% (w/v) nonfat dry milk. One-hundred microliters of the diluted supernatants was used for the sandwich ELISA analysis. Additionally, 200 μL of filtered supernatants were used for DNA extraction. One flask containing uninfected LMH cells was included as a negative control for each day of evaluation.

#### Embryonated SPF chicken eggs (*in ovo*)

Two-hundred microliters containing 0.58 × 10^3^ median tissue culture infective dose (TCID_50_) of a previously propagated VFAR-043 strain was inoculated on chorioallantoic membranes (CAMs) of twelve 10-day-old embryonated SPF chicken eggs. The eggs were incubated at 37°C for 4 days. The allantoic fluids (AFs) of three eggs were collected daily from 48 to 120 h after inoculation. AFs was centrifuged at 10,000 × *g* for 30 min at 4°C and filtered using 0.22μm filters. The obtained samples were diluted 1:2 in 1% (w/v) nonfat dry Milk and 100 μL was used for sandwich ELISA. Additionally, 200 μL of filtered AFs were used for quantitative polymerase chain reaction (qPCR). One PBS-inoculated 10-day-old embryonated SPF chicken egg was included as a negative control for each day of evaluation.

#### SPF chickens (*in vivo*)

Three-hundred microliters containing 1.7 × 10^3^ TCID_50_ of VFAR-043 was inoculated by intranasal/ocular routes to eight 14-day-old SPF chickens, which were obtained from FARVET SPF SAC (Ica, Peru). Clinical signs of infection were recorded daily from 2 to 13 days after inoculation. The clinical signs were classified according to Fuchs *et al*., 2005 [20]. Tracheal swabs were collected daily from 2 to 13 days after inoculation. For sampling, the swabs were pre-wetted with 200 μL of PBS, 100 μL was used for sandwich ELISA and the remaining 100 μL for qPCR. Eight PBS-inoculated 14-day-old SPF chickens were included in the analysis as negative controls. This experiment was approved by the Institutional Ethics Committee of the Universidad Peruana Cayetano Heredia, Lima, Peru (SIDISI: 65330). All SPF chickens were euthanized 14 days after inoculation using cervical dislocation without anesthesia following the American Veterinary Medical Association (AVMA) guidelines [21].

#### Sandwich ELISA assay

MaxiSorp 96-well microplates (Sigma Aldrich Co., St Louis, Missouri, USA) were coated with 100 μl of IgY polyclonal antibody (120 μg/mL) in carbonate buffer (pH 9.6) and incubated at 4°C overnight. Then, the microplates were washed five times with 200 μL of PBS-T 0.05% and blocked with 3% (w/v) nonfat dry milk (Sigma Aldrich Co., St Louis, Missouri, USA) in PBS-T 0.05% for 1 h at RT. One-hundred microliters of each sample, obtained from VFAR-043 inoculated LMH cells, embryonated SPF chicken eggs, and SPF chickens was added to the microplates, which were incubated for 1 h at 37°C and then at 4°C overnight. The microplates were then washed five times and 100 μL of biotinylated monoclonal antibodies (2.5 μg/mL for 1F5G8, 5F1B4, 10B5E2 and 5 μg/mL for 2G4E7) in 1% (w/v) nonfat dry milk were added to the wells and incubated for 1 h at 37°C. After five washing steps, 100 μL of streptavidin conjugated to HRP (Cat. No. ab7403; Abcam, Cambridge, MA, USA) at a 1:15 000 dilution in 1% (w/v) nonfat dry milk was added and incubated at 37°C for 30 min under agitation at RT. Microplates were washed five times with PBS-T 0.05%, 100 μl of TMB substrate (Sigma Aldrich Co., St Louis, Missouri, USA) was added and the plates were incubated at RT for 20 min. The reaction was stopped by adding 50 μL of 2N H_2_SO_4_ and microplates were read with an EON microplate reader (Biotek, Winooski, VT, USA) at 450 nm.

#### DNA extraction and quantitative polymerase chain reaction (qPCR)

DNA extraction was carried out using the QIAamp MinElute Virus Spin Kit (QIAgen, Hilden, Mettmann, Germany) following the manufacturer’s instructions. qPCR based on SYBR Green I was performed using specific primers for the amplification of 132bp fragment of the ILTV glycoprotein B gene. The qPCR was performed according to a previous study [22].

All samples for qPCR were evaluated individually, except tracheal swab samples, which were pooling daily for each group. All samples, including tracheal swabs, were tested as technical triplicates. The results obtained from qPCR were expressed as log10 viral genome copy number/mL.

### Statistical analysis

All quantitative data were analyzed using GraphPad Prism 6.0 (GraphPad Software, La Jolla, CA, USA). For indirect ELISAs, the assays were performed as triplicates and optical density (OD) values were represented as the mean plus the standard deviation (SD). The cutoff was calculated as the mean OD value of negative controls + 3SD. A sample was considered positive when the OD value was higher than the cutoff and negative when the OD value was lower than the cutoff. Background noise correction was carried out by subtracting the OD of the blank from sample OD. Two-way ANOVA and Tukey’s post hoc test were used for antibody comparison analysis.

For sandwich ELISA assays, linear regressions for calibration curves were performed to quantify gG production using two-fold serial dilution (20–0.0001 μg/mL) of the ILTV gG recombinant protein in each sandwich ELISA assay. Media and standard deviation (SD) were calculated for LMH cell as well as for embryonated SPF chicken egg analyses. Corrected OD values were calculated by subtracting the OD given by the uninfected LMH cell supernatant or uninfected allantoic fluid. The Mann-Whitney U test was performed for SPF chicken analysis.

## Results

### Expression of ILTV gG recombinant protein using baculovirus

It was developed a recombinant baculovirus, which was able to produce secreted ILTV gG recombinant protein in infected insect cells. A unique reactive ~40 kDa-protein band was observed by western blot using both an anti-6x-His tag antibody and serum from ILTV-infected chickens (Fig 1B and 1C). This result slightly differs from the expected molecular weight calculated for the ILTV gG recombinant protein, 30.1 kDa. Indeed, protein analysis by SDS-PAGE followed by Coomassie staining after protein purification allowed us to identify a unique protein band at ~40 kDa which corresponded to the protein mentioned above (Fig 1D).

### Reactivity evaluation of monoclonal antibodies against the ILTV gG recombinant protein analyzed by western blot

**Fig 2:**
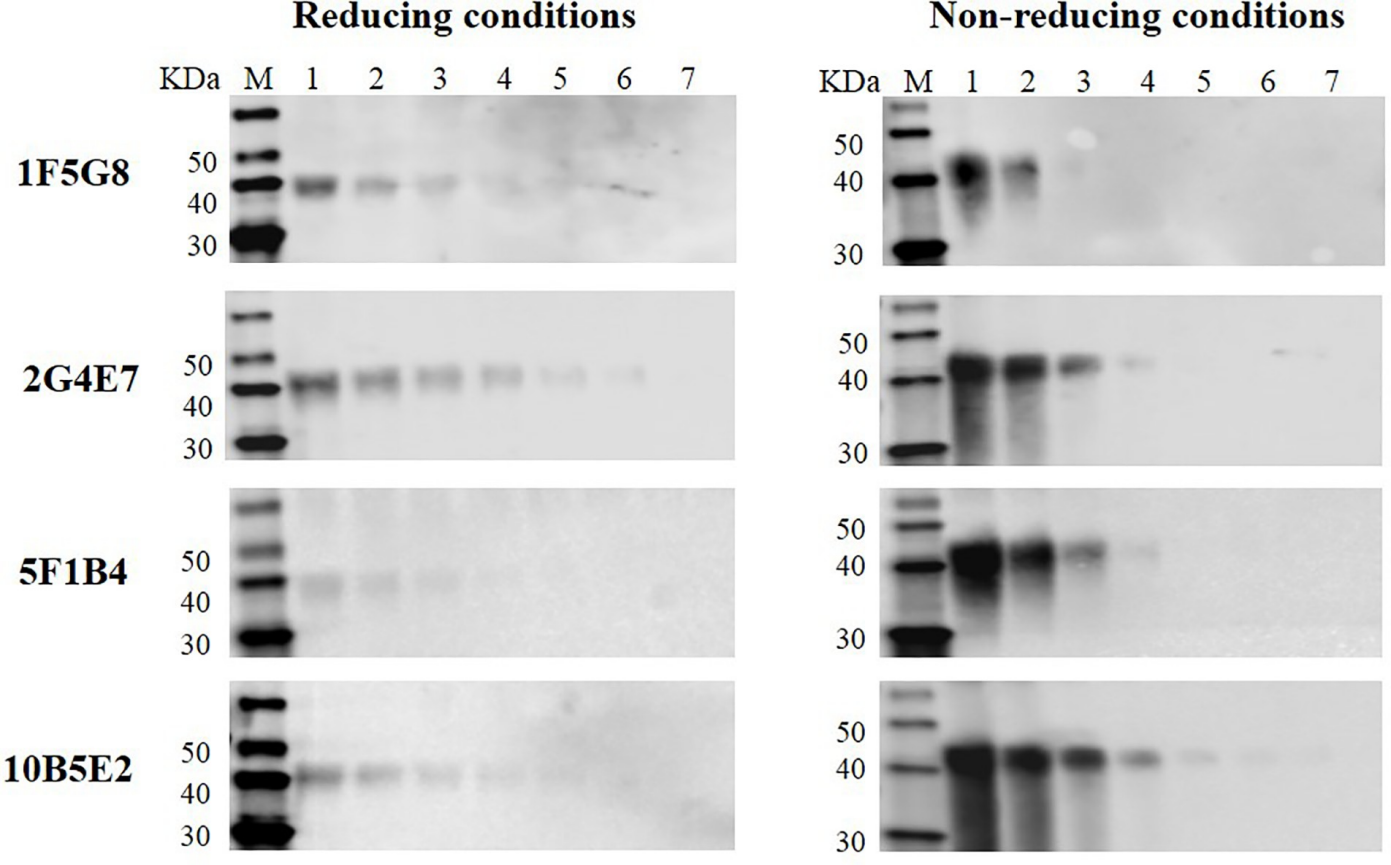
Identification of the purified ILTV gG recombinant protein by monoclonal antibodies (1F5G8, 2G4E7, 10B5E2, and 5F1B4) under reducing and non-reducing conditions in western blot. Lane M: Molecular weight marker (kDa). Two-fold serial dilution of the purified protein: lane 1: 1000 ng; lane 2: 500 ng; lane 3: 250 ng; lane 4: 125 ng; lane 5: 62.5 ng; lane 6: 31.25 ng; lane 7: 15.6 ng.

Four antibody-producing hybridoma clones were obtained and isotyped as follows: IgG2b (k) for 1F5G8, 2G4E7, and 10B5E2 clones, and IgG2a (k) for the 5F1B4 clone. The antibody titers were 4x10^-3^, 1x10^-4^, 5x10^-4^ and 8x10^-3^ for 1F5G8, 2G4E7, 10B5E2 and 5F1B4, respectively. All purified monoclonal antibodies were able to detect the ILTV gG recombinant protein. However, each antibody showed different levels of reactivity. 10B5E2 and 2G4E7 were the most reactive clones because they were able to detect the recombinant protein until 62.5 ng and 31.25 ng, respectively, in contrast to 1F5G8 and 5F1B4 which were the least reactive clones (until 125 ng) (Fig 2). All hybridoma clones generated during this study, namely 1F5G8, 2G4E7, 10B5E2, and 5F1B4, are available in the International Depositary Authority of Canada (IDAC) under accession numbers: 161018–01; 161018–02; 161018–03; 161018–04, respectively.

### Identification of the ILTV gG recombinant protein in an insect cell culture infected with recombinant baculovirus by immunofluorescence microscopy

**Fig 3:**
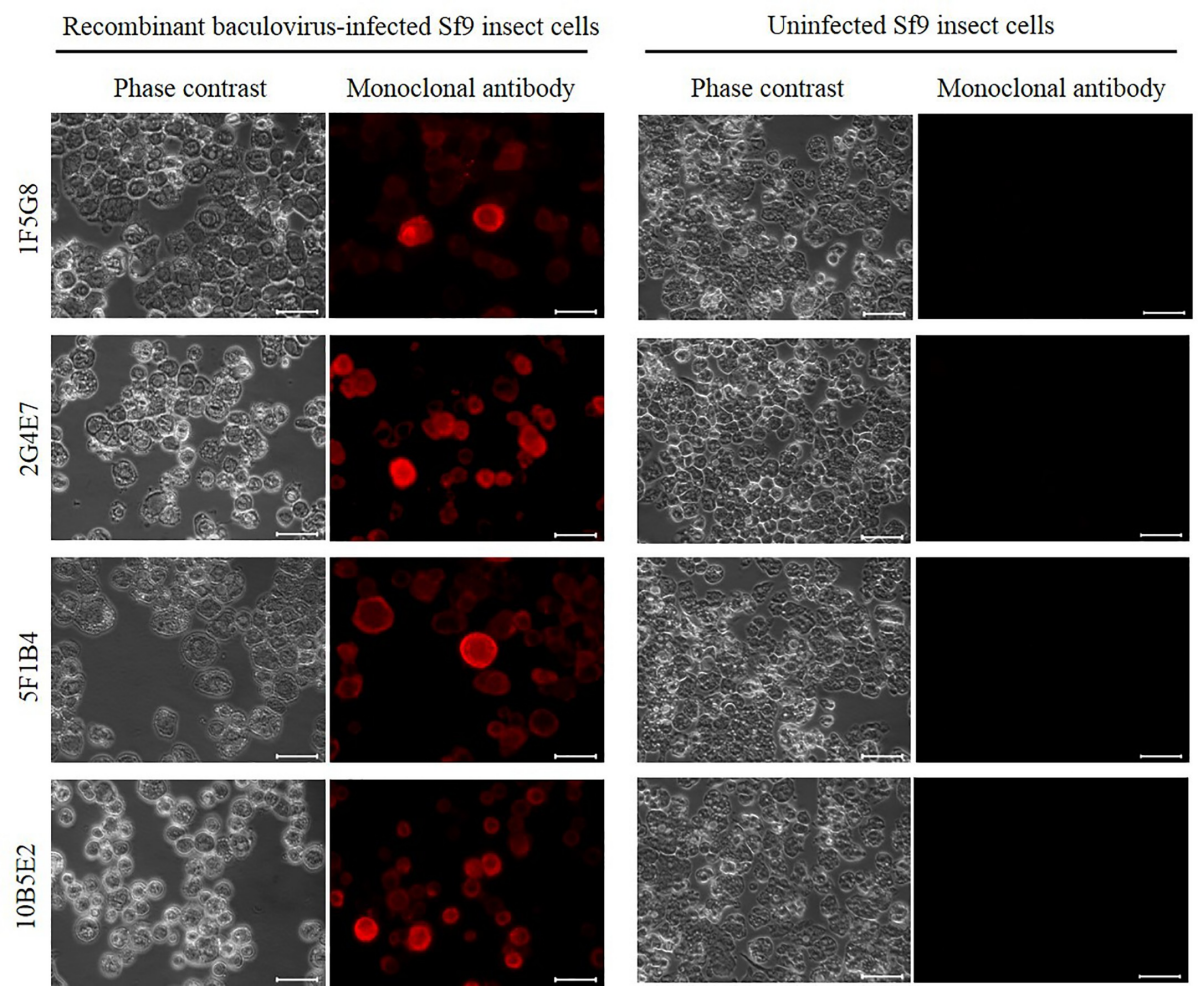
Monoclonal antibody recognition of the ILTV gG recombinant protein produced by a baculovirus expression vector system. Sf9 insect cells were infected with recombinant baculovirus and fixed at 72 h after infection. Immunofluorescence was performed with each anti-gG monoclonal antibody and with goat anti-mouse IgG antibody conjugated to Alexa Fluor 594. Red fluorescence corresponds to the binding of monoclonal antibodies to recombinant gG. Uninfected Sf9 cells were used as negative controls. Scale bars: 50 μm. Image magnification: 400x.

All monoclonal antibodies were able to identify ILTV gG expressed in recombinant baculovirus-infected Sf9 insect cells. In fact, the ILTV gG recombinant protein was identified in infected insect cell membranes, as expected for a secretory protein expression system. These infected cells showed a marked cytopathic effect in contrast to the negative control which showed a higher confluence (Fig 3).

### Identification of ILTV gG in an LMH cell culture infected with the Peruvian VFAR-043 strain using immunofluorescence microscopy

**Fig 4:**
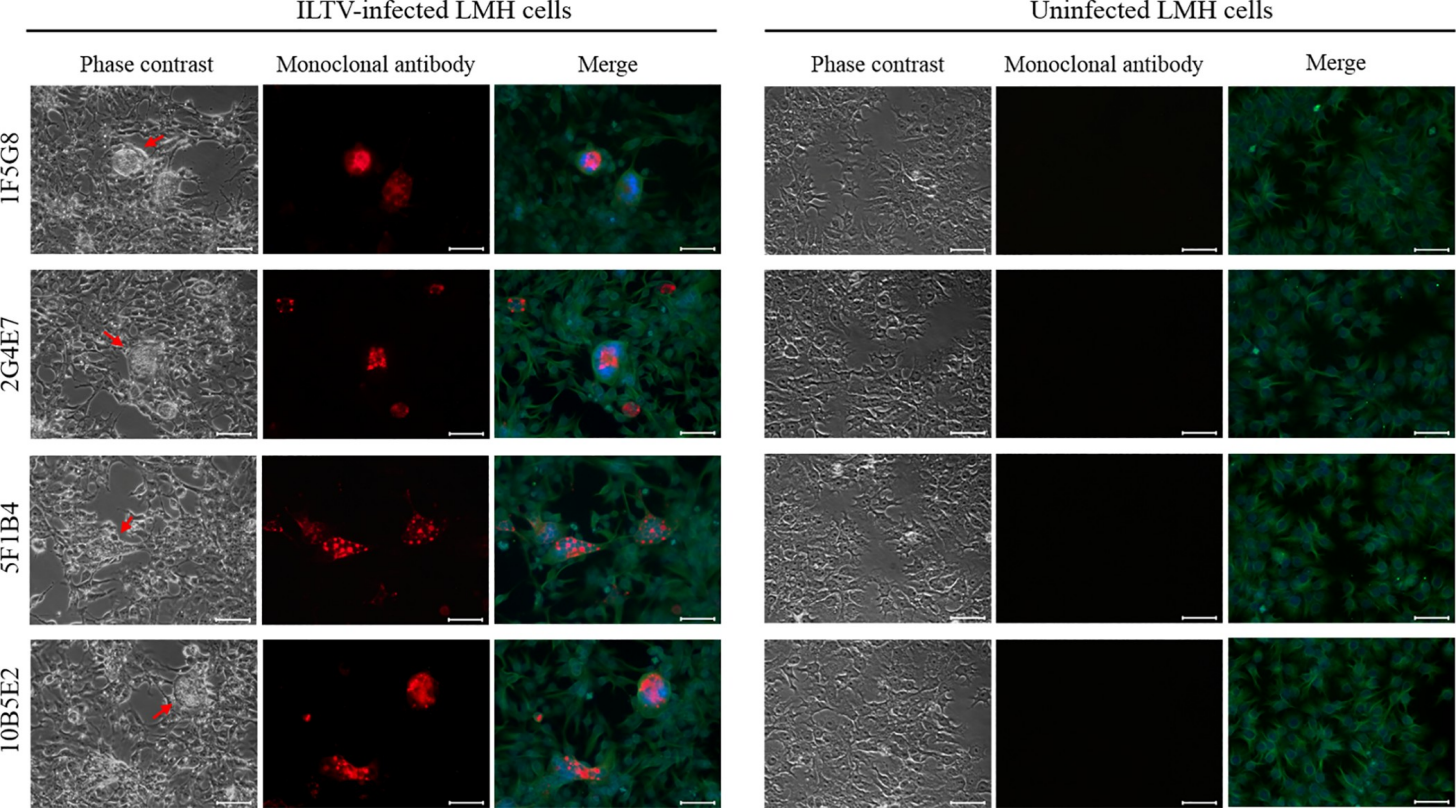
Recognition of gG by monoclonal antibodies in LMH cell cultures infected with the VFAR-043 strain. Immunofluorescence assays were carried out using each monoclonal and anti β-Tubulin III antibodies. Nuclear staining was performed with DAPI mounting solution. Green and blue fluorescence correspond to β-Tubulin III and nuclear staining, respectively. Uninfected LMH cells were used as negative controls. Red arrows show cytopathic effect zones. Scale bars: 50 μm. Image magnification: 400x.

We observed syncytia formations in LMH cells as a result of the ILTV infection. The presence of ILTV gG in these syncytia was detected by fluorescence microscopy using the purified monoclonal antibodies as primary antibodies and anti-rabbit IgG conjugated to Alexa Fluor 485 as a secondary antibody. On the other hand, no specific gG fluorescence signal was detected in uninfected LMH cells (negative control). These results showed the capability of our monoclonal antibodies to detect the target protein (ILTV gG) either as a recombinant protein in recombinant baculovirus-infected insect cells (Fig 3) or as a native protein in ILTV-infected LMH cells (Fig 4).

### Assessment of monoclonal antibodies using an indirect ELISA assay

**Fig 5:**
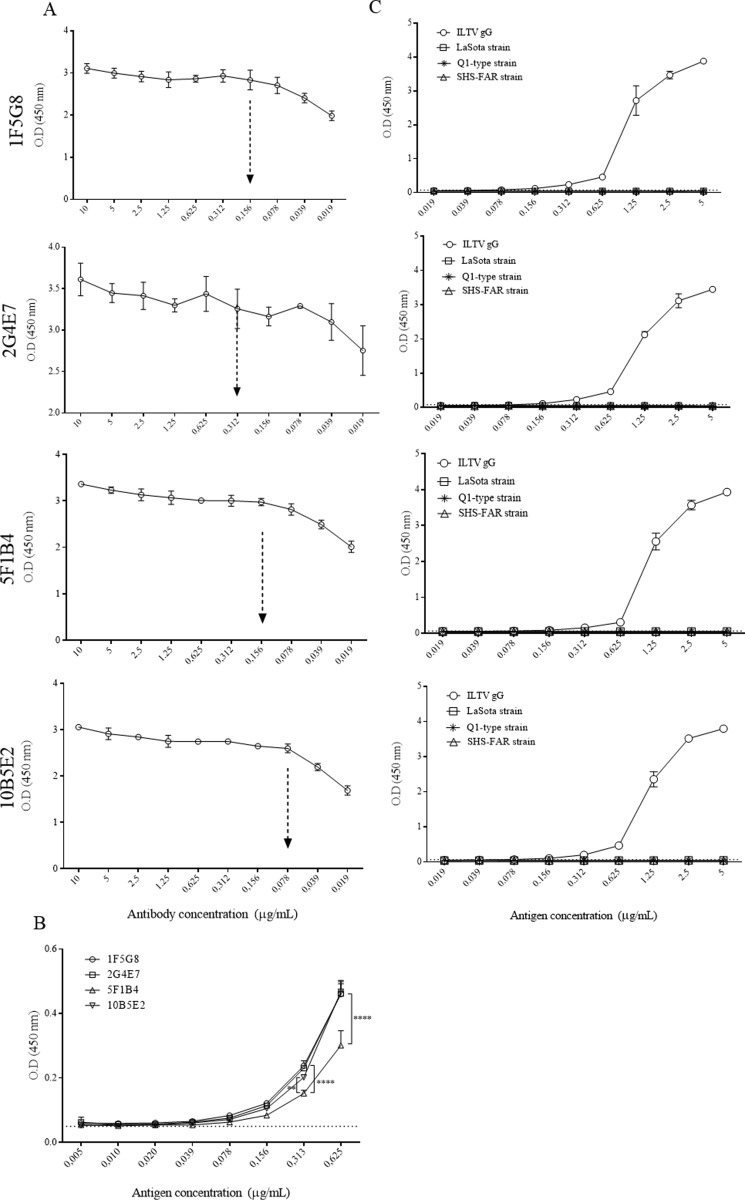
Evaluation of monoclonal antibodies using the ILTV gG recombinant protein in an indirect ELISA assay. A. Determination of the optimal monoclonal antibody concentration for the detection of ILTV gG. B. Limit of detection for each monoclonal antibody. C. Specificity evaluation for monoclonal antibodies using ILTV gG as a positive control and other related chicken respiratory viruses (Newcastle disease virus [LaSota strain], infectious bronchitis virus [Q1-type strain] and avian metapneumovirus [SHS-FAR strain]). Dashed arrows show the optimal antibody concentration. Cutoff = 0.05. Two-way ANOVA and Tukey’s post hoc were performed. **: p = 0.001. ****: p < 0.0001.

For the estimation of optimal antibody concentration, we selected the highest antibody concentration that showed non-saturating reactivity. The lowest antibody concentration (0.039 μg/mL) was detected for 2G4E7 and the highest (0.156 μg/mL) was detected for 1F5G8 and 5F1B4. A concentration of 0.078 μg/mL was detected for 10B5E2 (Fig 5A).

The limit of detection for each antibody was identified using the optimal antibody concentrations established above. The limit of detection was estimated as the lowest concentration of the recombinant ILTV gG detected above the cutoff. We identified that the limit of detection for all antibodies was 0.078 μg/mL (Fig 5B). Additionally, we observed that 5F1B4 showed lower reactivity, in comparison with other antibodies, at antigen concentrations of 0.625 and 0.313 μg/mL (p<0.0001).

For specificity evaluation, each monoclonal antibody was evaluated against other related chicken respiratory viruses such as the Newcastle disease virus, the infectious bronchitis virus and the avian metapneumovirus. No cross-reactivity was observed for any monoclonal antibody (Fig 5C).

### Assessment of monoclonal antibodies using sandwich ELISA assay

**Fig 6:**
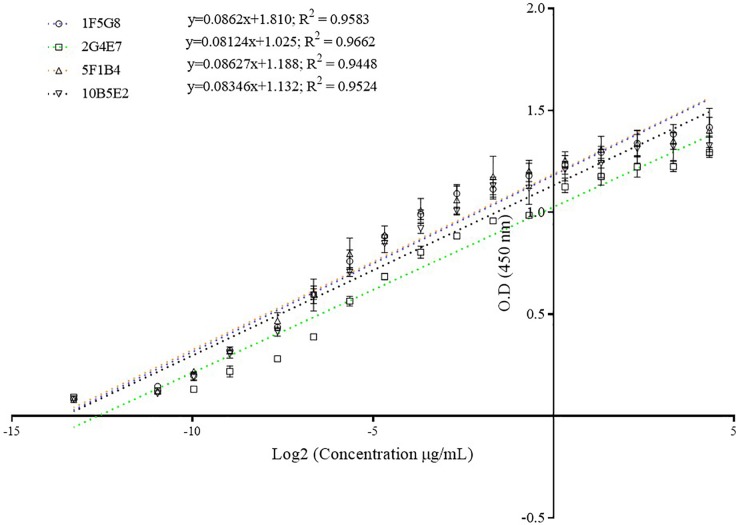
Calibration curves for each monoclonal antibody in a sandwich ELISA assay. The graphic shows the linear curves between OD and Log2 (ILTV gG recombinant protein concentration [μg/mL]). The equations and correlation coefficient (R^2^) are shown for each evaluated monoclonal antibody.

Four calibration curves were obtained for each evaluated monoclonal antibody. The equations as well as the correlation coefficients of the obtained calibration curves were y = 0.0862x + 1.810, R^2^ = 0.9583 for 1F5G8; y = 0.08124x + 1.025, R^2^ = 0.9662 for 2G4E7; y = 0.08627x + 1.188, R^2^ = 0.9448 for 5F1B4, and y = 0.08346x + 1.132, R^2^ = 0.9524 for 10B5E2 (Fig 6).

**Fig 7:**
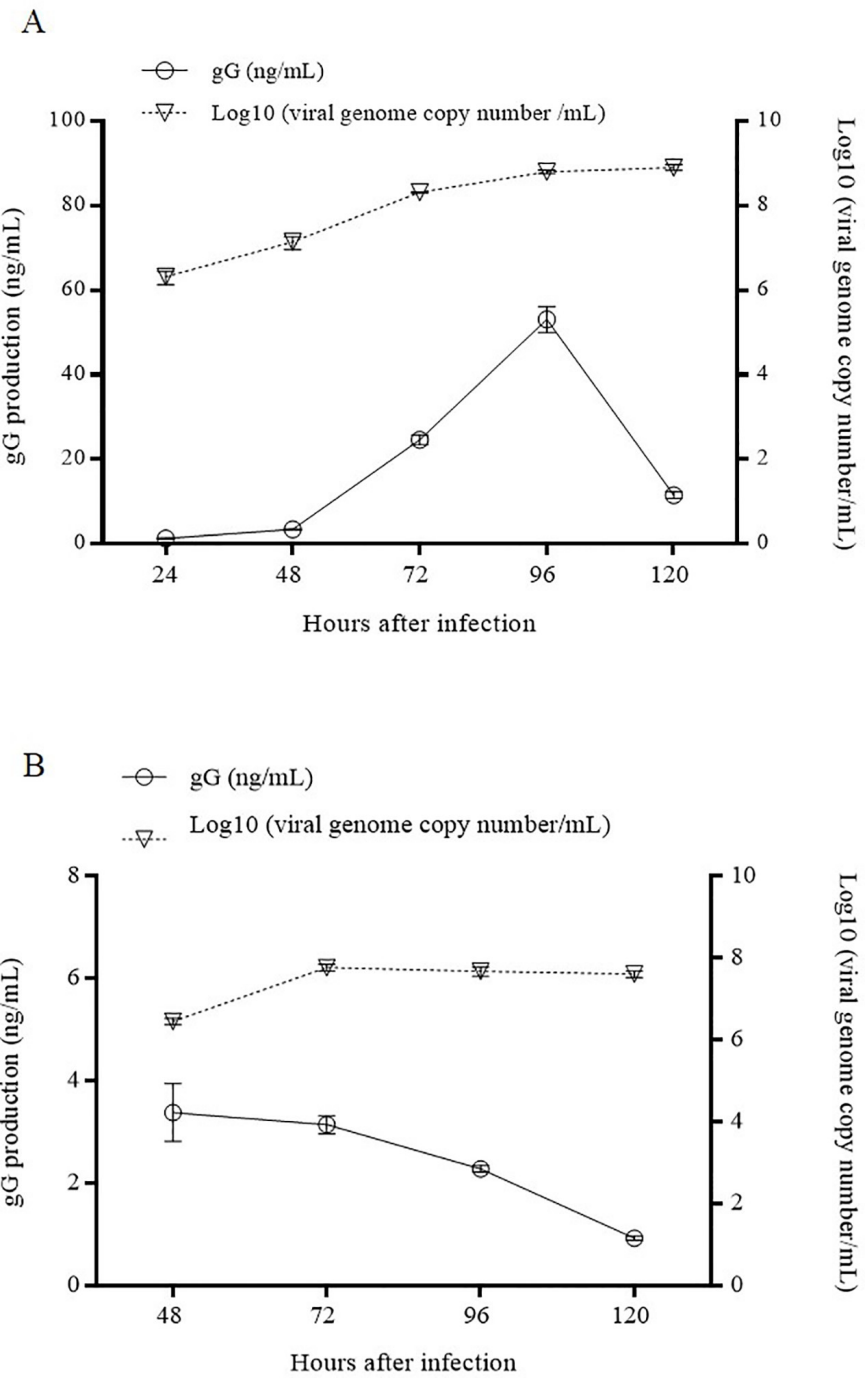
**gG production profile in LMH cells (A) and embryonated SPF chicken eggs (B) inoculated with VFAR-043.** gG production (ng/mL) was daily quantified by sandwich ELISA up to 120 hours after inoculation (Y left axis). Viral genome copy number was daily quantified by qPCR up to 120 hours after inoculation (Y right axis).

For LMH cells inoculated with VFAR-043, a gG-production peak (53.12 ± 3.1 ng/mL) was detected 96 h after inoculation, whereas a gradual increase in the ILTV genome copy number was observed up to 120 h after inoculation (Fig 7A). On the other hand, higher production of gG (3.39 ± 0.56 ng/mL) was observed 48 h after inoculation in embryonated SPF chicken eggs. While gG production gradually decreased until 120 h after inoculation, a peak was observed at 72 h after inoculation for the ILTV genome copy number, which gradually decreased until 120 h after inoculation (Fig 7B).

**Fig 8:**
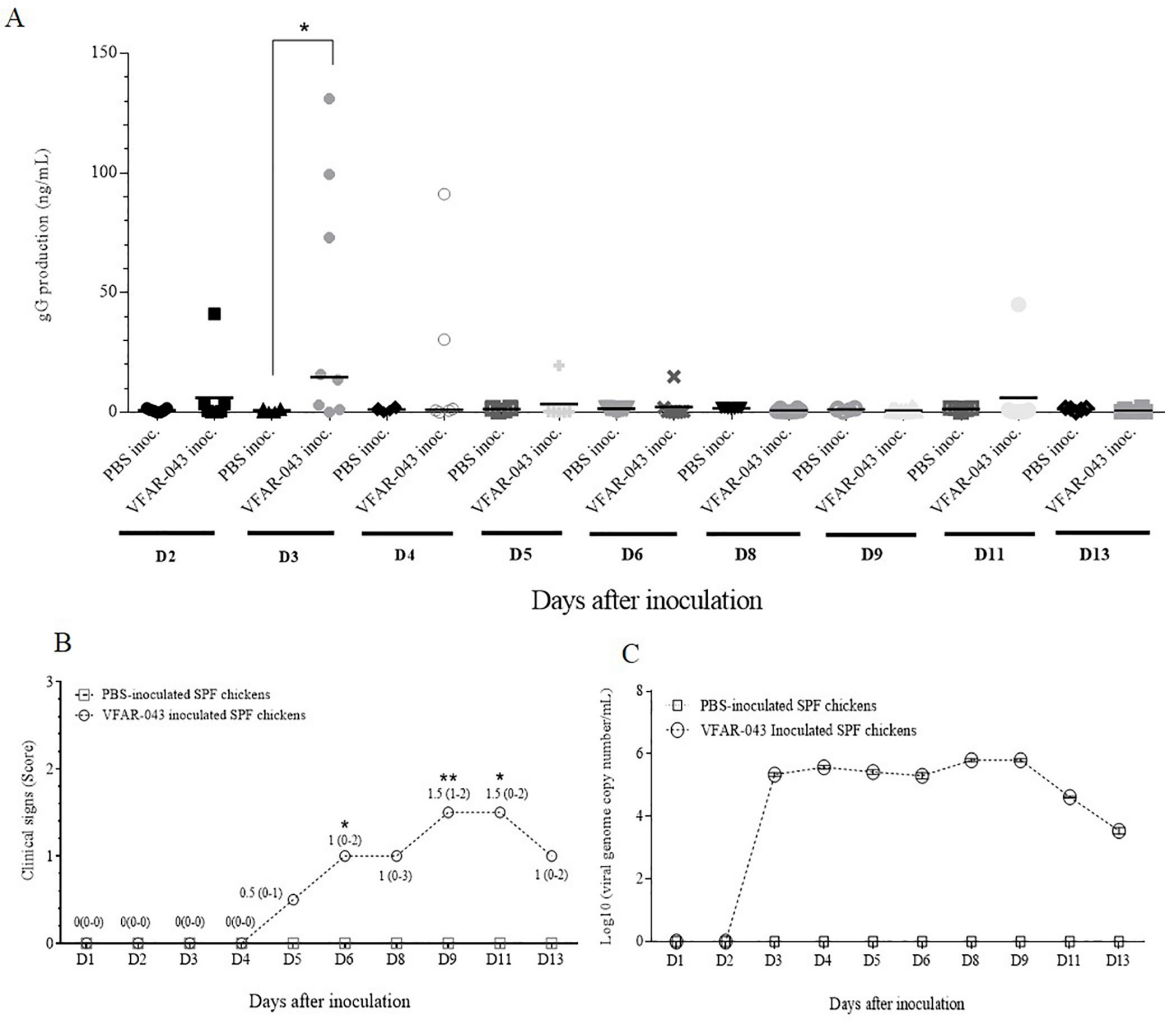
**Relationship among the gG production profile in tracheas (A), the clinical sign scores (B) and viral genome copy number in tracheas (C) from VFAR-043 inoculated SPF chickens.** PBS inoc.: PBS-inoculated SPF chickens; VFAR-043 inoc.: VFAR-043 inoculated SPF chickens. (A): the horizontal lines indicate the median value for each group. (B): only numerical values for medians and ranges are shown for VFAR-043 inoculated SPF chicken and all medians for PBS-inoculated SPF chickens were zero. (C): Each point represents daily sample pooling for each group. All values were expressed as a log10 (viral genome copy numbers/mL). The Mann–Whitney U test was performed for analyses. * = p<0.05; ** = p<0.005.

In the SPF chicken evaluation, a high gG production peak was detected on day 3 after VFAR-043 inoculation in SPF chickens (14.75 ng/mL [0.01–131.10]) in comparison to PBS-inoculated SPF chickens (0.58 ng/mL [0.12–1.73]) (p = 0.0426) (Fig 8A). Additionally, higher clinical sign scores as well as a gradual increase of ILTV genome copies were observed 9–11 (Fig 8B) and 3–9 (Fig 8C) days after VFAR-043 inoculation, respectively, in SPF chickens compared to the PBS-inoculated control.

## Discussion

The viral chemokine binding proteins (vCKBPs) have been associated with viral immunomodulatory mechanisms in their respective hosts [10, 23]. In the alphaherpesvirus family, gG is considered a vCKBP owing to its affinity to chemokines. gG is a secreted protein produced in most members of this family, including animal and human alphaherpesviruses, with the exception of the varicella zoster virus (VZV) and the Marek’s disease virus [10]. The immunological role of gG in chickens after infection with ILTV has been well described, but its production profile during ILTV infection remains poorly known.

In order to study the gG production profile, we successfully expressed an ILTV gG recombinant protein in the Sf9 insect cell culture (Figs 1, 2 and 3). This allowed us to generate 4 monoclonal antibodies. All monoclonal antibodies were able to detect the ILTV gG recombinant protein in western blot analyses under reducing or non-reducing conditions, demonstrating their promising use for further diagnostic tool development.

Similar to our results, a previous study reported the expression of ~40kDa-ILTV gG recombinant protein in Sf9 insect cells [13]; however, the theoretical molecular weight calculated for this protein in this study was 30.9 kDa. Moreover, the presence of ILTV gG with a higher molecular weight from ILTV infected cells has been attributed to the addition of carbohydrates at N-linked glycosylation sites [7]. Thus, this could explain the detection of higher molecular weight protein in this study.

In contrast to other studies, where the identification of gG was carried out using polyclonal antibodies during western blot [5, 7, 12, 13], this study developed monoclonal antibodies that were also able to detect gG in syncytia formed by ILTV infection in LMH cell cultures (Fig 4). A similar pattern was also observed in monolayer primary cells infected with ILTV on semi-solid medium [12]. The results from both studies suggest that agar-containing media trap gG in formed syncytia, facilitating its identification.

ELISA assays are widely used for detection of analytes, but their use requires monoclonal antibody development. In this study, three out of four developed monoclonal antibodies reached similar reactivity when the antibody limit of detection was evaluated using indirect ELISA (Fig 5B). In order to quantify gG in LMH cell cultures, embryonated SPF chicken eggs, and SPF chickens inoculated with VFAR-043, we developed a quantitative ELISA assay in a sandwich format using the developed monoclonal antibodies. The capability of monoclonal antibodies to detect the target of interest was evaluated through calibration curves. The highest correlation coefficient (R^2^ = 0.9662) was observed for 2G4E7, and this antibody was selected for further evaluations. The gG production profile after infection with ILTV has not been extensively studied and the detection of gG in ILTV-infected cell cultures had only been performed using polyclonal antibodies [5, 7, 12, 13]. We consider that the evaluation of gG production profiles could deepen the knowledge of viral mechanisms in the host, as gG is considered to play a crucial role in the viral immunomodulation of chicken immune response [9]. We found that the gG production profiles in ILTV-infected cell cultures and embryonated SPF chicken eggs were slightly different. While the highest gG production in infected culture cells was 53.12 ± 3.1 ng/mL at 96 h after inoculation, the highest production of gG in embryonated SPF chicken eggs was 3.39 ± 0.56 ng/mL at 48 h after inoculation (Fig 7). This gG production difference could be attributed to the extracellular accumulation of gG in cell cultures, facilitating its detection, in contrast to the possible interaction of gG with other molecules, such as chemokines, hampering its detection by monoclonal antibodies in eggs. Moreover, the low gG production in embryonated SPF chicken eggs could be related to the gG distribution in other embryonic tissues different from allantoic fluid or gG degradation carried out in infected chicken embryos.

On the other hand, qPCR was used to evaluate the viral genome copy number regardless active or inactive viral particles. We observed that the number of viral genome copies increased progressively after ILTV infection in cell cultures, even when gG production had declined (Fig 7). Furthermore, we detected a remarkable increase in the number of viral genome copies at 72 h post-inoculation in embryonated SPF chicken eggs, while the highest gG production was observed 48 h after infection. This finding suggests that viral replication, as evidence of genome copy number increase, initiates after gG production in ILTV-inoculated embryonated SPF chicken eggs.

A similar pattern was detected in VFAR-043-inoculated SPF chicken tracheas, where a peak of gG production was observed earlier than the appearance of clinical signs. While other studies reported the onset of clinical signs on day 3 after inoculation with field strains [24, 25], we detected the appearance of clinical signs on day 5 after inoculation with VFAR-043 strain, which is a strain phylogenetically related to two U.S strains belonging to genotype VI [26]. Moreover, Oldoni and colleagues reported the onset of clinical signs for these genotype VI strains on day 3 after inoculation [27], in contrast to the results obtained in this study. Thus, a more extensive investigation into genotype VI field strains are necessary in order to clarify these discrepancies including evaluation of inoculation route and chicken lineages.

Interestingly, the gG production peak and a gradual increase of viral genome copy were detected initially on day 3 after inoculation. These two events occurred before the clinical signs appearance on day 5 in VFAR-043 infected SPF chickens.

All these results contribute to knowledge of the gG role during the ILTV infection, reinforcing the importance of gG as a virulence factor, which has been described to be capable of modulating chicken immune response [13] and promoting cell-to-cell transmission of ILTV in the host [12]. Likewise, its early production emphasizes that gG could be a potential candidate for early detection of ILTV infection through the development of rapid diagnostic tools such as immunochromatographic assays targeting this secreted glycoprotein. Finally, it should be taken into consideration that this study shows the gG production profile using the VFAR-043 strain; which is phylogenetically related to two US field strains [26]; therefore, the evaluation of the gG profile using other ILTV strains, including chicken embryo origin (CEO) and tissue culture origin (TCO), could greatly differ, and further studies will be required to determine the gG profile during infection in other ILTV strains.

Finally, a limitation of this study was to use the pooling of sample for each group analysis of SPF chicken to evaluate the viral genome copies by qPCR. Therefore, we recommend evaluating individually and in details each sample that belongs to a given experimental group.
